# A New Inertial Aid Method for High Dynamic Compass Signal Tracking Based on a Nonlinear Tracking Differentiator

**DOI:** 10.3390/s120607634

**Published:** 2012-06-07

**Authors:** Yao Guo, Wenqi Wu, Kanghua Tang

**Affiliations:** College of Mechanical Engineering and Automation, National University of Defense Technology, Changsha 410073, Hunan, China; E-Mails: wenqiwu_lit@hotmail.com (W.W.); tt_kanghua@hotmail.com (K.T.)

**Keywords:** high dynamics, Compass/INS integrated navigation system, inertial aiding, nonlinear tracking differentiator

## Abstract

In Compass/INS integrated navigation systems, feedback inertial navigation solutions to baseband tracking loops may eliminate receiver dynamic effects, and effectively improve the tracking accuracy and sensitivity. In the conventional inertially-aided tracking loop, the satellite-receiver line-of-sight velocity is used directly to adjust local carrier frequency. However, if the inertial solution drifts, the phase tracking error will be enlarged. By using Kalman filter based carrier phase tracking loop, this paper introduces a new inertial aid method, in which the line-of-sight jerk obtained from inertial acceleration by a nonlinear tracking differentiator is used to adjust relevant parameters of the Kalman filter's process noise matrix. Validation is achieved through high dynamic Compass B3 signal with line-of-sight jerk of 10 g/s collected by a GNSS simulator. Experimental results indicate that the new inertial aid method proposed in this paper is free of the impact of the receiver dynamic and inertial errors. Therefore, when the integrated navigation system is starting or re-tracking after losing lock, the inertial error is absent from the navigation solution correction that induces large drift, and the new aid method proposed in this paper can track highly dynamic signals.

## 1. Introduction

The new Beidou (Compass) Navigation Satellite System network, consisting of five GEO satellites and 30 non-GEO satellites, is designed to provide positioning service with an accuracy of 10 m, speed measurement service with an accuracy of 0.2 m/s and time service with an accuracy of 10 ns within the whole global area. By the end of April 2012, 13 navigation satellites will have been launched. Some signal parameters of Compass were publicized in the 2010 Munich Satellite Navigation Summit [1]. With the establishment of the Compass network and the demand for current high dynamic applications such as spaceflight and aviation, research on Compass/INS integrated navigation system has been gradually receiving more intensive attention. In the Compass/INS integration system, INS measurements and Compass measurements are integrated using Kalman filtering to prevent inertial solution drift and also to aid the baseband tracking loop by adjusting local signal generation.

The receiver's baseband tracking loop, normally, consists of a phase locked loop (PLL) for carrier tracking and a delay locked loop (DLL) for code tracking. Generally, the receiver adopts a third-order PLL, and loop parameters are determined with the controlled-root method [2]. Moreover, the PLL based on Kalman filtering has also been widely used. It is possible for the Kalman-PLL to adjust the loop gain according to the discriminator output, achieving adjustment of loop bandwidth, effectively reducing the loop's response time and restraining tracking noise [3,4]. In a weak signal and highly dynamic environment, the PLL without assistance can easily lose lock [5–7]. However, the integration system feeds the inertial measurements to the tracking loop, so the tracking error caused by receiver dynamics can be eliminated with the aid of inertial information. Therefore, the loop's bandwidth will be reduced and its tracking precision will be improved. Two factors which are seldom concerned need to be considered when using inertial measurements to aid highly dynamic tracking loops:

1. In a highly dynamic situation, the inertial sensor's bias will be increased. For instance, the bias of an accelerometer with a ±100 g measuring range is approximately 0.1 g to 1 g [8–10]. Under such conditions, will the inertial aid still be efficient?
2. If the integrated navigation system is starting or re-tracking, or the satellite signal is interfered or blocked, the inertial solution error fails to be corrected without the help of integration output. Under such conditions, will the inertial aid still be efficient?

These problems will both result in large offsets or drifts in the inertial aid information. Since the code tracking loop is less affected by receiver dynamics, and its dynamic influence can be overcame through carrier frequency aiding, our research emphasis shall be given to inertially-aided PLL.

Nowadays most GNSS/INS integrated navigation systems based on inertially aided baseband tracking are in the form of tightly integration or ultra-tightly integration [11]. In the 2000s, the GPS/INS ultra-tightly integration system was designed and applied [12]. The difference between tight integration and ultra-tight integration is that, the tight integration is a cascade of baseband filters and navigation filters, whereas the ultra-tight integration is implemented in a single filter based on the estimation of baseband signal measurement errors [13,14]. Ultra-tight integration may eliminate the time-correlated noise of cascaded filters to improve the tracking sensitivity and navigation precision [15]. Herein, according to the baseband of Compass/INS tightly integration, inertially-aided Kalman-PLL is adopted. Carrier phase, Doppler frequency shift, and Doppler shift rate are taken as state vectors, and the output of PLL discriminator is used to adjust the Kalman filter gain dynamically. The Doppler shift rate noise is relative to satellite-receiver line-of-sight jerk. Normally a small value is taken for the system noise covariance matrix **Q** [3], thus **Q** will have large error in a highly dynamic situation with large line-of-sight jerk, and a traditional Kalman-PLL will result in divergence. The inertial velocity and acceleration measurements could help to correct the relevant values of the Kalman states to a great extent, but if there is large offset in the aid information, Kalman-PLL will result in filter divergence.

Hence, it is necessary to consider the influence of Doppler shift rate variation. Doppler shift rate value is relative to line-of-sight acceleration, so line-of-sight jerk can be considered as the coefficient of Kalman states or noise. Considering that increasing Kalman states will accordingly increase the computation burden and large jerk normally has short duration (for jerk 30 g/s, only 2 s are needed to reach an acceleration of 60 g), this paper takes line-of-sight jerk as the process noise. Then it is feasible to consider a new inertial aid method, using the line-of sight jerk as aid information to adjust the noise coefficient of the Doppler shift rate state in real time.

Line-of-sight jerk can be acquired through time difference of line-of-sight acceleration calculated by inertial measurements and Compass ephemeris. The time difference method can eliminate the influence of acceleration offset or drift induced by INS bias, but the line-of-sight jerk acquired by the time differentiator will have a large noise. The nonlinear tracking differentiator presented by Han Jingqing [16] in 1994 can implement the difference of any signal, and it has great filtering capacity. Nonlinear tracking differentiator has been efficiently applied to various fields, such as satellite navigation [17], magnetic suspension [18] and signal processing [19]. This paper refers to this method and its improved versions [20] for achieving smoothed line-of-sight jerk through the inertial acceleration with noise, and efficiently aiding high dynamic Compass signal tracking.

## 2. Inertial Aided PLL in Compass/INS Integrated Navigation System

**Figure 1.**
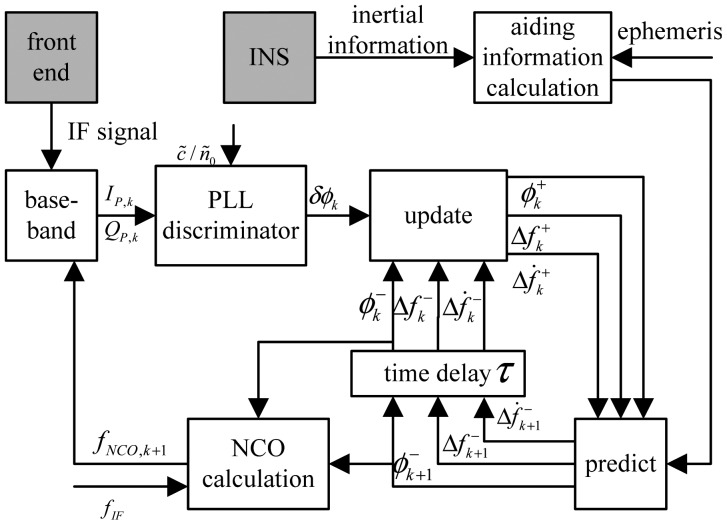
Inertial aided PLL in Compass/INS integrated navigation system.

The inertial aided Kalman-PLL is adopted in the Compass/INS integrated navigation system. With carrier phase, Doppler frequency shift, and Doppler shift rate as the system states, carrier phase as the observation, and phase discriminator output as the innovation, the carrier phase and Doppler frequency shift are estimated and used to compute the carrier NCO, as shown in Figure 1 [21].

The system matrix of Kalman-PLL is demonstrated as:

(1) $$\left[\begin{array}{c} \phi_{k+1}\\ \Delta f_{k+1}\\ \Delta {\dot{f}}_{k+1} \end{array}\right]=\left[\begin{array}{ccc} 1 & 2\pi \tau & \pi \tau^{2}\\ 0 & 1 & \tau \\ 0 & 0 & 1 \end{array}\right]\left[\begin{array}{c} \phi_{k}\\ \Delta f_{k}\\ \Delta {\dot{f}}_{k} \end{array}\right]+\left[\begin{array}{c} 2\pi f_{IF}\tau \\ 0\\ 0 \end{array}\right]+{\text{W}}_{K+1}$$

where *τ* is the loop update interval. The process noises are approximated as white noises:

(2) $$\begin{array}{l} E(w_{k})=0\\ E(w_{k}w_{k}^{T})=Q_{k} \end{array}$$

where the covariance matrix of the process noise **Q***_k_* is related to receiver's dynamic characteristics.

The measurement matrix of Kalman-PLL is demonstrated as:

(3) $$\phi_{k}=[\begin{array}{ccc} 1 & 0 & 0 \end{array}]\left[\begin{array}{c} \phi_{k}\\ \Delta f_{k}\\ \Delta {\dot{f}}_{k} \end{array}\right]+{\text{V}}_{k}$$

Observation noises are also approximated as white noises:

(4) $$\begin{array}{c} E(v_{k})=0\\ E(v_{k}v_{k}^{T})={\text{R}}_{k} \end{array}$$

The covariance matrix of observation noise **R***_k_* is determined by carrier phase tracking noise due to thermal noise, vibration noise, ionosphere scintillation, noise of oscillator and other sources.

Adopt an arctan phase discriminator, and the output of phase discriminator is demonstrated as:

(5) $$\delta \phi_{k}=\text{atan}(Q_{p}/I_{p})$$

where, *Q_p_*, *I_p_* are the quadra-phase output and in-phase output of baseband correlator prompt channel.

Take the PLL discriminator output *δφ_k_* as the observation innovation, that is:

(6) $$Z_{k/k-1}=\delta \phi_{k}$$

The computation of Kalman-PLL is presented with two steps, prediction and update:

Prediction:

(7) $$\begin{array}{l} {\text{x}}_{\text{k}+1}^{-}=\Phi_{\text{k}}{\text{x}}_{\text{k}}^{+}\\ {\text{P}}_{\text{k}+1}^{-}=\Phi_{\text{k}}{\text{P}}_{\text{k}}^{+}\Phi_{\text{k}}^{\text{T}}+\Gamma_{\text{k}}{\text{Q}}_{\text{k}}\Gamma_{\text{k}}^{\text{T}} \end{array}$$

Update:

(8) $$\begin{array}{l} {\text{K}}_{\text{k}}={\text{P}}_{\text{k}}^{-}{\text{H}}_{\text{k}}^{\text{T}}{\left({\text{H}}_{\text{k}}{\text{P}}_{\text{k}}^{-}{\text{H}}_{\text{k}}^{\text{T}}+{\text{R}}_{\text{k}}\right)}^{-1},\\ {\text{x}}_{\text{k}}^{+}={\text{x}}_{\text{k}}^{-}+{\text{K}}_{\text{k}}({\text{Z}}_{\text{k}}-{\text{H}}_{\text{k}}{\text{X}}_{\text{k}}^{-})={\text{x}}_{\text{k}}^{-}+{\text{K}}_{\text{k}}Z_{k/k-1},\\ {\text{P}}_{\text{k}}^{+}=(\text{I}-{\text{K}}_{\text{k}}{\text{H}}_{\text{k}}){\text{P}}_{\text{k}}^{-}{\left(\text{I}-{\text{K}}_{\text{k}}{\text{H}}_{\text{k}}\right)}^{\text{T}}+{\text{K}}_{\text{k}}{\text{R}}_{k}{\text{K}}_{k}^{\text{T}}) \end{array}$$

where **K**_k_ is the optimal gain matrix.

The carrier NCO is:

(9) $$f_{\text{NCO},k+1}=\frac{\phi_{k+1}^{-}-\phi_{k}^{-}}{2\pi \tau }$$

where, *φ_k_* is the carrier phase, Δ*f_k_* is the Doppler frequency shift, and Δ*ḟ_k_* is the Doppler shift rate. The superscript “+” denotes the updated measurement and the superscript “−” denotes the predicted measurement.

The essential part of inertial aided tracking is to use the inertial aid information to predict the Doppler frequency shift, and then eliminate the loop's dynamic stress error. The traditional aid method is velocity aiding, and acceleration aiding is also practical [22].

The velocity aid method involves computing the Doppler frequency shift through the line-of-sight velocity, and correcting the estimated Doppler frequency shift before the update step (8), as shown in Equation (10):

(10) $$\Delta {\hat{f}}_{k}=\frac{f_{ca}}{c}{\text{v}}_{k}{\text{e}}_{k}$$

Acceleration aiding involves computing the Doppler shift rate through the line-of-sight acceleration, and correcting the estimated Doppler shift rate before the update step (8), as shown in Equation (11):

(11) $$\Delta {\hat{\dot{f}}}_{k}=\frac{f_{c\text{a}}}{c}a_{k}{\text{e}}_{k}$$

where **v**_k_ is the line-of-sight velocity vector, **a**_k_ is the line-of-sight acceleration vector, and **e**_k_ is line-of-sight unit vector. In a highly dynamic situation with satellite-receiver line-of-sight jerk, if there is large offset or drift in inertial aiding information, the aided Kalman-PLL will diverge.

## 3. Inertial Aiding Method Based on a Nonlinear Tracking Differentiator

### 3.1. Jerk Aided Kalman-PLL

The process noise covariance matrix **Q**_k_ of Kalman-PLL is demonstrated as [21]:

(12) $${\text{Q}}_{k}=\left[\begin{array}{ccc} 0 & 0 & 0\\ 0 & 0 & 0\\ 0 & 0 & n_{\Delta \ddot{f},k}^{2}\tau \end{array}\right]$$

where $n_{\Delta \ddot{f}}^{2}$ denotes the Doppler shift rate PSD, that is:

(13) $$n_{\Delta \ddot{f},k}^{2}=\sigma^{2}(\Delta {\dot{f}}_{k}-\Delta {\dot{f}}_{k-1})/\tau$$

The Doppler shift rate value is related to satellite-receiver line-of-sight acceleration. Parameters of process noise covariance matrix **Q**_k_ are related to the line-of-sight jerk. When the line-of-sight jerk is constant or it varies slowly, $n_{\Delta \ddot{f}}^{2}$ can be set as a very small constant. However, if the line-of-sight jerk is large, the process noise characteristic is unknown, and filtering divergence might occur. There are two solutions to such a problem: the first one is to estimate the receiver's dynamic characteristics through adaptive algorithms, and adjust the coefficients of process noise covariance matrix [23,24]; the second one is to introduce inertial aiding information [25], and carry out real time correction of the process noise covariance matrix during the prediction step.

The Doppler shift rate is related to satellite-receiver line-of-sight acceleration, is demonstrated as:

(14) $$\begin{array}{cc} n_{\Delta \ddot{f},k}^{2} & =\sigma^{2}(\Delta {\dot{f}}_{k}-\Delta {\dot{f}}_{k-1})/\tau \\ & =\frac{\sigma^{2}(\frac{f_{c\text{a}}}{c}\cdot {\text{a}}_{k}-\frac{f_{ca}}{c}\cdot a_{k-1})}{\tau }\\ & =\sigma^{2}(\frac{f_{ca}}{c}\cdot {\text{j}}_{k})\tau \end{array}$$

where *f_ca_* is the carrier frequency, *c* is the light velocity, **a**_k_ is the satellite-receiver line-of-sight acceleration vector.

The jerk aided PLL involves performing time difference of line-of-sight acceleration, obtaining the line-of-sight jerk, and correcting the process noise matrix relevant coefficient $n_{\Delta \ddot{f},k}^{2}$ of the Kalman-PLL according to Equations (12) and (14).

### 3.2. Jerk Aided Kalman-PLL Based on Nonlinear Tracking Differentiator

If the input signal with noise is *y*(*t*), its differential *x*(*t*) can be deduced by a time differential method. Generally the time differential method is as follows:

(15) $$x(t)=\frac{y(t)-y(t-\tau )}{\tau }$$

The above equation matches Equation (14), and this differential operation will increase the noise. Using the nonlinear tracking differentiator, the smoothed output *y˜*(*t*) and its differential *x*(*t*) can be obtained through the input *y*(*t*), and the equation is as the following:

(16) $$\{\begin{array}{c} \tilde{y}(t+\tau )=\tilde{y}(t)+\tau \cdot \tilde{x}(t)\\ \tilde{x}(t+\tau )=\tilde{x}(t)+\tau \cdot f(e,\tilde{x}(t))\\ e=\tilde{y}(t)-y(t) \end{array}$$

where *τ* is sampling interval, *f*(·) is nonlinear function. The function *f*(·) used herein is [26]:

(17) $$f(e,\tilde{x}(t))={\text{R}}^{2}\left[-{\text{a}}_{0}e-{\text{a}}_{1}\text{fal}(e,\alpha ,\delta )-{\text{a}}_{2}\text{fal}(\frac{\tilde{x}(t)}{\text{R}},\alpha ,\delta )\right]$$

where the function fal(·) is expressed as [26]:

(18) $$\text{fal}(x,\alpha ,\delta )=\{\begin{array}{c} x^{\alpha }\text{sign}(x),\left|x\right|\geq \delta \\ x/\delta^{1-\alpha },\left|x\right|\leq \delta \end{array}$$

If R is larger, the tracking is faster, but the noise will be accordingly increased. The nearer α approaches 1, the more the system approaches linearity. The setting of threshold value *δ* is in order to prevent the system from vibrating in the origin, and *δ* should be far less than 1. Here, we select the parameters as follows: set T as per sampling frequency and R = 2, a_0_ = 60, a_1_ = 60, a_2_ = 60, α = 6/9.

### 3.3. Data Verification of Nonlinear Tracking Differentiator

**Figure 2.**
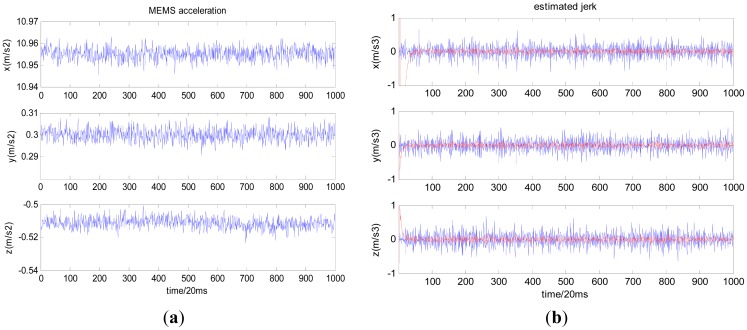
Jerk estimated from the MEMS acceleration with the two methods (**a**) MEMS acceleration; (**b**) Jerk estimated with the two methods.

Firstly, let us take the Micro-Electromechanical System (MEMS) acceleration data collected under static conditions as an example (Figure 2(a)), and estimate the jerk with a time differentiator and nonlinear tracking differentiator, respectively; the results are shown in Figure 2(b) where the blue curve stands for the jerk obtained with the time differentiator and the red curve stands for the jerk obtained with the nonlinear tracking differentiator. Table 1 shows the offset and noise standard deviation (std) of estimated jerk under static conditions with the two methods.

**Figure 3.**
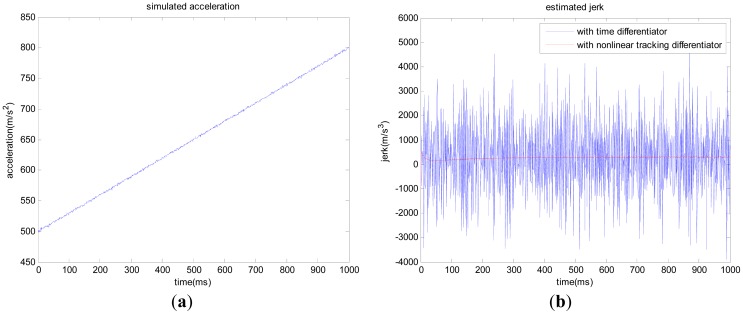
Jerk estimated from the simulated high dynamic acceleration with the two methods (**a**) Simulated high dynamic acceleration; (**b**) Jerk estimated with the two methods.

Secondly, let us take the simulated line-of-sight acceleration with 50 g to 80 g variation within one second as an example (Figure 3(a)). The offset of simulated acceleration is 0.1 g, and the noise standard deviation is 0.1 g. We compare the jerk obtained with the time differentiator and nonlinear tracking differentiator, respectively, and the results are shown in Figure 3(b). Table 2 shows the offset and noise standard deviation (std) of estimated jerk in the high dynamic condition with the two methods.

As shown in Figures 2(b) and 3(b), the nonlinear tracking differentiator can overcome the time differentiator's disadvantage of enlarging noise and time delay. For Compass/INS integrated navigation system, if the following situation occurs:

1. Because the inertial measurement unit with wide measuring range has large bias, the acceleration aiding information will have a constant offset;
2. When the integrated navigation system is starting, or the satellite signal is interfered or blocked, the integrated navigation output will be halted. The inertial error will fail to be corrected, and the inertial error will be enlarged gradually with time.

Under general conditions, the third-order PLL or traditional inertially-aided PLL can track the satellite signal with any line-of-sight acceleration. If the above situation occurs and the receiver-satellite line-of-sight jerk is large, it is feasible to track the high dynamic signal with the proposed jerk aided PLL, *i.e.*, estimate the line-of-sight jerk through inertial acceleration based on a nonlinear tracking differentiator, and adjust the process noise matrix coefficients of the Kalman-PLL.

## 4. Experiments

Due to the limited hardware conditions and difficulties in collecting actual high dynamic satellite signals and synchronous inertial measurement data, it is optimal to use: (1) the high dynamic simulated intermediate frequency data generated by Matlab; (2) the high dynamic data collected based on GNSS signal simulator to verify the proposed aiding methods.

### 4.1. Simulation Test Based on Matlab

First of all, we use the simulated high dynamic GNSS intermediate frequency data generated by Matlab and synchronous line-of-sight aiding data to verify the PLL performance with the following three aid methods and make a comparison:

Aid method 1: PLL without assistance
Aid method 2: acceleration aided PLL
Aid method 3: jerk aided PLL based on a nonlinear tracking differentiator

The above three aid methods are all based on Kalman-PLL. The acceleration-aided PLL involves adjusting the value of Kalman states according to Equation (11), and the jerk aided PLL method involves adjusting the process noise matrix coefficients of the Kalman filter according to Section 3.

**Figure 4.**
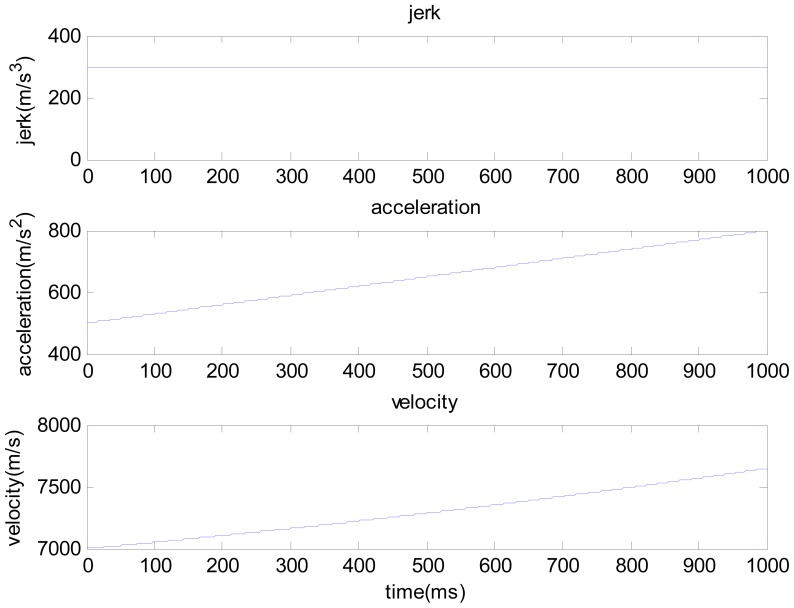
The high dynamic characteristics of simulated signal (line-of-sight direction).

**Figure 5.**
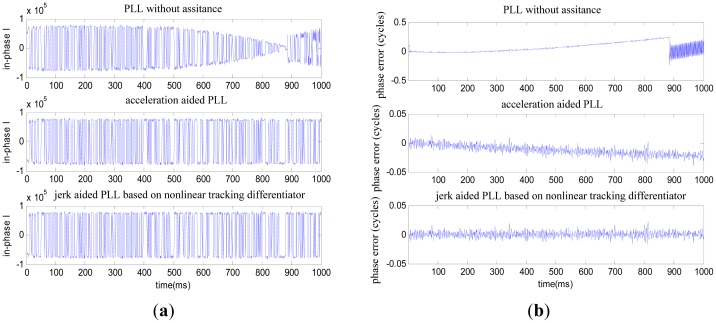
(**a**) Comparison of the in-phase output; (**b**) Comparison of the tracking errors.

**Figure 6.**
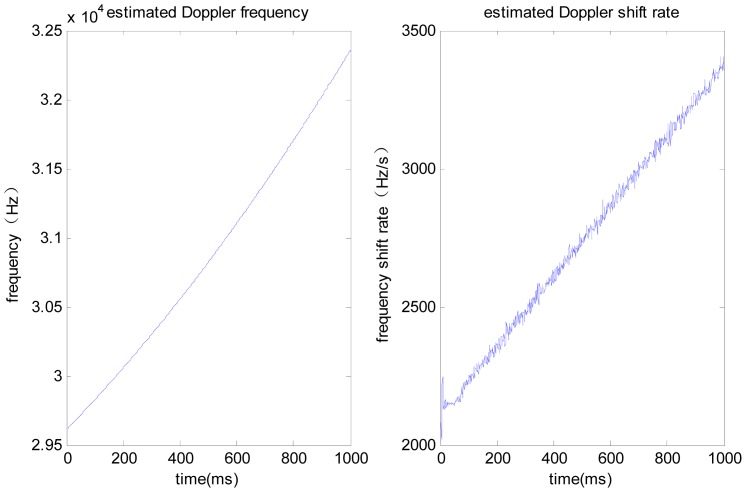
Estimated Doppler frequency and Doppler shift rate with jerk aided PLL.

The one second duration high dynamic intermediate frequency data produced by the Matlab satellite signal simulator is used here, and the SNR is −15 dB. As shown in Figure 4, the receiver's dynamic characteristic is: the initial line-of-sight velocity is 7,000 m/s, initial line-of-sight acceleration is 50 g and jerk is constant 30 g/s. Consider the conditions of aiding line-of-sight acceleration error as: the line-of-sight acceleration offset is 1 g [8–10], noise standard deviation is 0.1 g. See Figures 5 and 6 for the tracking results.

As shown in Figure 5, where the line-of-sight acceleration bias is 1 g, noise standard deviation is 0.1 g, the PLL without assistance will fail to track; the acceleration-aided PLL can track in short duration scenarios, but long-duration tracking precision will be lowered; the jerk-aided PLL can track stably. As shown in Figure 6, the Doppler frequency estimated with the jerk-aided PLL is about 29,700 to 32,300 Hz, and the estimated Doppler shift rate is about 2,200 to 3,400 Hz/s, which matches with the receiver's dynamic characteristics.

It is concluded on the above experimental results of simulated high dynamic signal that if there is a large offset or drift in the inertial aid information, the jerk-aided PLL based on a nonlinear tracking differentiator can be efficiently used to track the Compass B3 signal with line-of-sight jerk of 30 g/s.

## 4.2. Test Based on GNSS Signal Simulator

**Figure 7.**
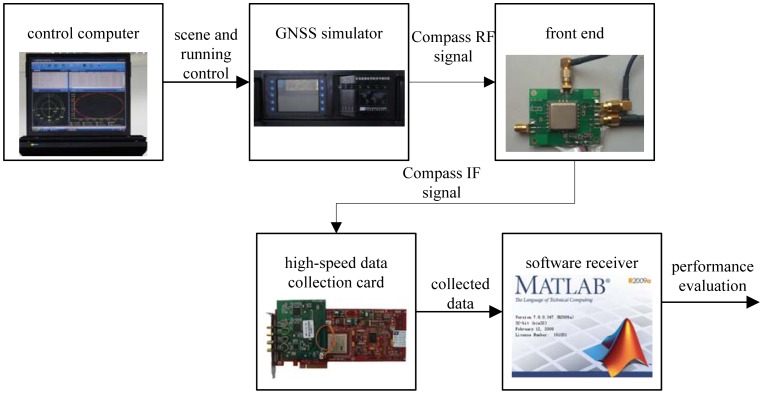
The test system composition based on GNSS simulator.

The next used the Compass B3 signal collected with the GNSS signal simulator to verify the proposed aid method. The experimental facility consists of a control computer, a GNSS signal simulator, a radio frequency front end and a high-speed data collection card, as illustrated in Figure 7. The GNSS signal simulator can output radio-frequency signals of various constellations. The control computer provides the GNSS signal simulator with the functions of scene editing, downloading and running state monitoring (including simulation time, satellites state, and user trajectory). Users can customize scenes according to their necessary receiver dynamic characteristics. It also provides special scenes with satellite-to-receiver relative motion. After the radio-frequency signal collection, we implement intermediate frequency signal acquisition and tracking in a Matlab based software receiver.

**Figure 8.**
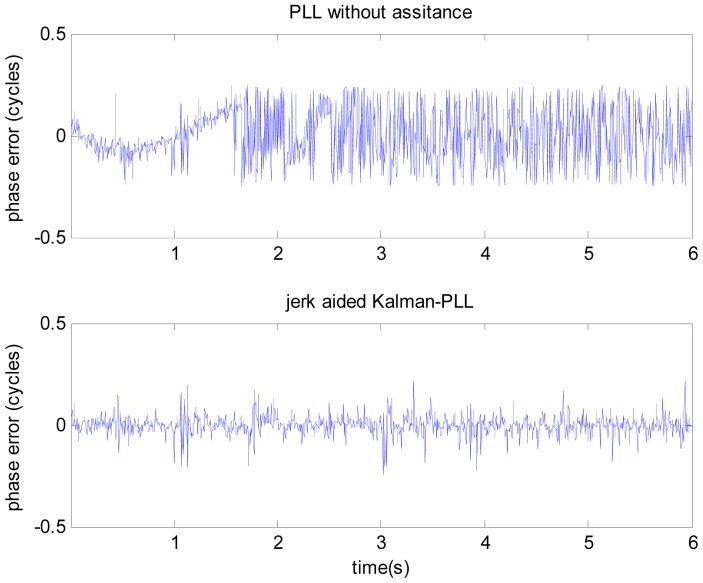
Phase tracking errors comparison of GNSS simulator collected signal.

**Figure 9.**
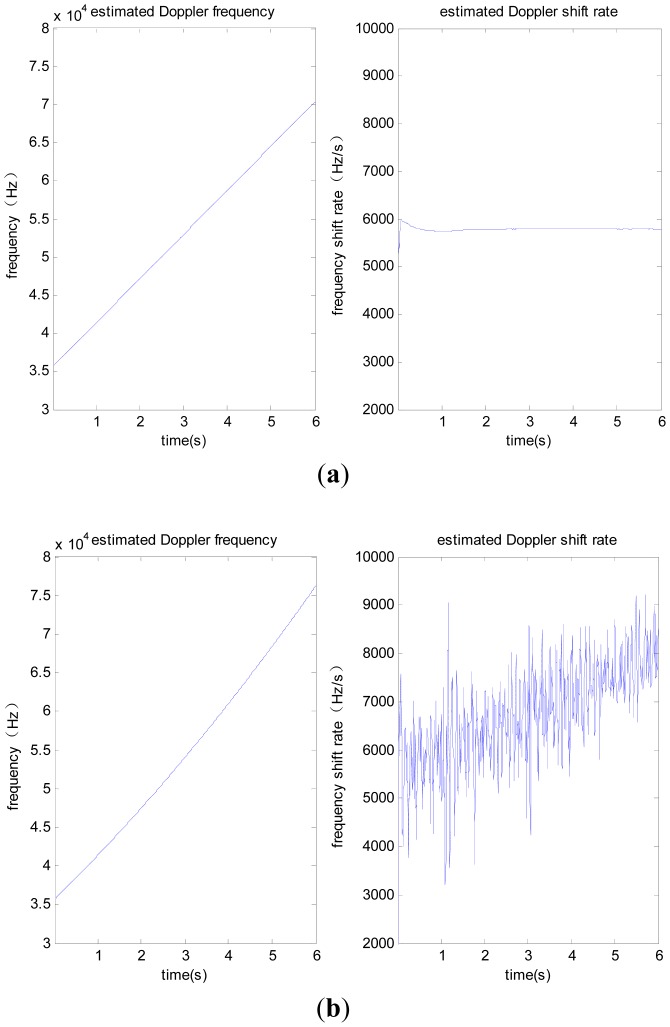
(**a**) Doppler frequency and Doppler shift rate estimated by PLL without assistance; (**b**) Doppler frequency and Doppler shift rate estimated by jerk aided Kalman-PLL.

The test adopts the scene of satellite-to-receiver relative motion, with the initial line-of-sight velocity of 8,000 m/s, initial acceleration of 100 g, constant jerk of 10 g/s, signal power of −110 dBm. We collect intermediate frequency data for a duration of 6 seconds. Taking the PRN 6 satellite signal as an example, it is difficult to obtain the correct line-of-sight acceleration, so the jerk-aided PLL based on the nonlinear tracking differentiator is verified according to the constant line-of-sight jerk of 10 g/s. The performance of jerk-aided Kalman-PLL is compared with the PLL without assistance. The results are shown in Figures 8 and 9.

Figure 8 shows the comparison of carrier phase tracking errors. It is concluded that the PLL without assistance fails to track, but the jerk-aided Kalman-PLL can perform stable tracking with a phase tracking error of about 13°. As shown in Figure 9, the Doppler frequency and frequency shift rate that are estimated by the PLL without assistance cannot reflect real dynamic characteristics, however, the values estimated by jerk-aided Kalman-PLL match with the receiver dynamic characteristics.

From the test results based on the GNSS simulator, the jerk-aided Kalman-PLL can efficiently track the highly dynamic GNSS signal with constant line-of-sight jerk of 10 g/s. Meanwhile, it is concluded from Figures 2 and 3 that high precision line-of-sight jerk can be estimated with the nonlinear tracking differentiator. In result, the high dynamic Compass B3 signal can be efficiently tracked with jerk-aided Kalman-PLL in the Compass/INS integrated navigation system, by using the inertial acceleration measurements to estimate line-of-sight jerk based on the nonlinear tracking differentiator, and adjusting relevant coefficients of the process noise matrix of the Kalman-PLL.

## 5. Conclusions

This paper introduces a jerk-aided Kalman-PLL in the Compass/INS integrated navigation system, where the jerk is estimated through inertial acceleration measurements based on the nonlinear tracking differentiator. This aid method can eliminate the impact of inertial error on the aided tracking loop. Based on the test of high dynamic Compass B3 signals collected by the GNSS simulator, it is concluded that the proposed new aid method can efficiently help track highly dynamic signals with 10 g/s line-of-sight jerk. In conclusion, if the Compass/INS integrated navigation system is starting or re-tracking after losing lock, the inertial error correction by integrated navigation solution hasn't been implemented, then the jerk-aided Kalman-PLL based on the nonlinear tracking differentiator as presented herein can work efficiently.

## Figures and Tables

**Table 1.** Offset and noise standard deviation of estimated jerk in static condition with the two methods.

	**Estimated jerk by time differentiator (m/s^2^)**			**Estimated jerk by nonlinear tracking differentiator (m/s^2^)**		

	**x**	**y**	**z**	**x**	**y**	**z**
offset	0.0001	0.0002	0.0001	0.00003	0.00004	−0.00008
std	0.1911	0.1870	0.2026	0.0565	0.0567	0.0619

**Table 2.** Offset and noise standard deviation of estimated jerk in high dynamic condition with the two methods.

	**Estimated jerk by time differentiator (m/s^2^)**	**Estimated jerk by nonlinear tracking differentiator (m/s^2^)**
offset	2.7396	0.0059
std	1,359.8	13.7883
